# *Mixta mediterraneensis* as a novel and abundant gut symbiont of the allergen-producing domestic mite *Blomia tropicalis*

**DOI:** 10.1007/s10493-023-00875-3

**Published:** 2024-01-16

**Authors:** Tomas Erban, Bruno Sopko, Pavel B. Klimov, Jan Hubert

**Affiliations:** 1 Crop Research Institute, Drnovska 507/73, 161 06 Prague 6 – Ruzyne, Czechia; 2 Purdue University, Lilly Hall of Life Sciences, G-225, 915 W State St, West Lafayette, IN 47907 USA; 3 Department of Microbiology, Nutrition and Dietetics, Faculty of Agrobiology, Food and Natural Resources, Czech University of Life Sciences Prague, Kamycka 129, 165 00 Prague 6 – Suchdol, Czechia

**Keywords:** Mite, Allergen, Digestion, Enzyme, Symbionts

## Abstract

*Blomia tropicalis* is an allergen-producing mite in the human environment in tropical regions. The microbiome of *B. tropicalis* was described using the barcode sequencing region of V4 16S rDNA and genome assemblage. *Mixta mediterraneensis*, previously isolated from human skin swabs, was identified as a *B. tropicalis* gut symbiont based on genome assembly. The microbiome contains two bacteria, *Staphylococcus* and *M. mediterraneensis*. The number of *M. mediterraneensis* 16S DNA copies was 10^6^ per mite and 10^9^ per feces in the rearing chamber based on qPCR quantification. The profile of this bacterium reached 50% of reads in the mite gut and feces. Genomic analyses revealed that the bacterium has several metabolic pathways that suggest metabolic cooperation with the mite host in vitamin and amino acid synthesis, nitrogen recycling, and antimicrobial defense. Lysozyme is present in the symbiotic bacterium but absent in the mite. The *B. tropicalis* microbiome contained *Staphylococcus*, which accelerates mite population growth. Mites can digest *Staphylococcus* by using specific enzymes with hydrolytic functions against bacterial cell walls (chitinases and cathepsin D), leading to endocytosis of bacteria and their degradation in lysosomes and phagosomes. Gene expression analysis of *B. tropicalis* indicated that phagocytosis was mediated by the PI3-kinase/Akt pathway interacting with the invasins produced by *M. mediterraneensis*. Moreover, the symbiont had metabolic pathways that allowed it to recycle the mite metabolic waste product guanine, known as a mite attractant. The mite host symbiont enhances mite aggregation in the feces, and the fecal–oral transmission route is excepted.

**Supplementary Information:**

The online version contains supplementary material available at 10.1007/s10493-023-00875-3.

## Introduction

*Blomia tropicalis* Van Bronswijk, de Cock & Oshima (Acari: Astigmata: Echimyopodidae) (van Bronswijk et al. 1974), is commonly present in house dust in tropical and subtropical regions (Vrtala 2022), feeding on dust, skin, nail particles and microorganisms inhabiting these substrates (Colloff 2009). To date, 21 allergen groups with different biological functions have been described in this mite (Vrtala 2022). However, despite direct or indirect relevance to the allergenic properties of mites, the interactions of *B. tropicalis* with associated bacteria are poorly understood. In other house dust mites, bacterial symbionts, such as *Cardinium* and *Bartonella*-like species, can modulate the expression of allergen-encoding genes and produce endotoxins that promote or prevent an allergic response (Erban et al. 2020a; Kaur et al. 2021; Valerio et al. 2005). Associated symbiotic bacteria may form a nutritional symbiosis, enhancing mite digestion through various mechanisms, e.g., vitamin provision and nitrogen recycling (Erban et al. 2016b). Previous studies revealed that *B. tropicalis* extracts contain endotoxins (Barboza et al. 2013), probably originating from an abundant, gram-negative bacterium belonging to the family Erwiniaceae (Hubert et al. 2016). Unfortunately, this bacterium has not been characterized further.

Erwiniaceae is a species-rich family of gram-negative bacteria that are rod-shaped, facultatively anaerobic and non-spore-forming (Soutar and Stavrinides 2022). This family includes bacteria of the genus *Pantoea* and *Mixta*, which are plant pests (Li et al. 2017; Walterson and Stavrinides 2015), insect symbionts inhabiting the digestive systems of their hosts (Karamipour et al. 2016; Kashkouli et al. 2021; Walterson and Stavrinides 2015), and nitrogen fixers in fungal gardens of leaf-cutting ants (Pinto-Tomas et al. 2009). For example, *Pantoea* bacteria are abundant members of the gut microbial community and include larvae and adults of herbivorous insects, e.g., *Spodoptera litoralis* (Chen et al. 2016), *Plutella xylostella* (Li et al. 2017), *Tetreaponera pilosa* (Stoll et al. 2007), stink bug *Halyomorpha halys* (Kenyon et al. 2015) and *Rhagoletis mendax* (Maccollom et al. 2009). Some insect associates show a genome reduction trend, for example, from 3.9 to 5 Mb in free-living bacteria to 0.8–2.8 Mb in endosymbiotic bacteria (Kashkouli et al. 2021). The association of *B. tropicalis* with an Erwiniaceae bacterium is the only mite-bacterium association identified to date (Hubert et al. 2016); however, this association has not been characterized, and it may involve biologically important interactions.

Here, we characterized the microbiome of the allergen-producing mite *B. tropicalis* based on V4 16S rRNA sequencing and identified *Staphylococcus* and Erwiniaceae bacteria as the only abundant sequences in the mite microbiome. Based on the mite metagenomic DNA samples, we assembled the genome of Erwiniaceae bacteria. The next comparison of the assembled genome revealed that it belongs to *Mixta mediterraneensis* according to high similarity to *M. mediterraneensis* strain Marseille-Q2057^T^ that was previously isolated from skin swabs from the hand of a 30-year-old healthy woman (Boxberger et al. 2021). We focused on describing interactions between mites and bacterial symbionts using functional metagenomics, meta-transcriptomics, and proteomics. Comparison results suggested possible mutualistic interactions of identified bacteria and with the acarine host.

## Materials and methods

### Samples of mites and feces

The culture of *B. tropicalis* originated from rearing facilities of the Crop Research Institute, Prague, Czechia, and was maintained as described previously (Hubert et al. 2019). Cultivation was performed in Iwaki flasks on a house dust mite diet (HDMd) (Erban and Hubert 2008) composed of dog kernels (Ontario-pet, Placek, Podebrady, Czechia), wheat germ, aqua-tropic—dried fish food (Lon-Bio, Praha, Czechia), Mauripan dried yeast extract (AB Mauri, Hampton, Peterborough, UK), and gelatin (SERVA Electrophoresis, Heidelberg, Germany) (ratio 10:10:3:1 wt). The mixture was powdered and sieved and heated in a thermostat (70 °C) for 0.5 h to suppress microbial growth. The mites were collected with a brush into sterile tubes and weighed. Feces were collected from the flasks, and residual mites or eggs were then removed (Erban and Hubert 2015). For the experiments, we weighed 30–40 mg of mites using a microbalance (Metler-Toledo). Surface sterilization was performed on ice. The mite surfaces were cleaned by placing them in 100% ethanol, followed by vortexing for 5 s and centrifugation at 13,000×*g* for 1 min. The supernatant was replaced with a 1:10 bleach solution containing 5% sodium hypochlorite, and the samples were then mixed by vortexing for 5 s and centrifuged at 13,000×*g* for 2 min. The bleach was replaced by ddH_2_O, and this step was repeated twice to remove residual bleach. The rearing diets were sampled before mite addition. Samples of both rearing diets and mite feces were not further cleaned; after collection, they were stored in an ultracold freezer. The samples were taken in six biological replicates for barcode sequencing, five samples for RNA isolation and 1 sample of mites for mite genomic DNA isolation.

For the proteomic analysis, the following four sample types, each with three biological replicates, were prepared as described previously (Hubert et al. 2023): (i) 1,000 individually collected adult mites; (ii) pooled samples of mites at different developmental stages, including eggs; (iii) water extracts of feces; and (iv) detergent-buffer extracts of the remaining pellet of the water extract, obtained as described previously (Erban et al. 2016b, 2017; Erban and Hubert 2015; Hubert et al. 2023). Protein samples were transferred to the Proteomics Core Facility, BIOCEV, for analyses.

### Bacterial cultivation and identification of cultivated bacteria

Fecal samples were diluted in 3 mL of double distilled H_2_O, and the mixture was diluted (10^–1^ to 10^–6^) and plated on nutrient agar plates. After 24 h at 37 °C, the bacterial colonies were isolated and identified by PCR using the eubacterial 27F and 1492R and rpoB primers (Drancourt and Raoult 2002). The PCR products were purified, cloned and sequenced at Macrogen (Seoul, South Korea) following an established protocol (Hubert et al. 2012). Sequences were identified using BLASTn (Altschul et al. 1990; Benson et al. 2013) and deposited in GenBank (Accession Ids: KY865751, KY865752).

### Mite population growth on the *Staphylococcus* additive diet

Two *Staphylococcus* isolates were inoculated on brain heart infusion in 6 Falcon tubes and allowed to grow for 5 days. After 5 days, 5 mL of double distilled H_2_O was added to every tube, and the sample was mixed. The supernatant was transferred to a centrifugation tube and processed as described previously (Erban et al. 2016b). The HDMd rearing diet (Erban and Hubert 2008) was enriched with *Staphylococcus* at 0.001 and 0.0001% by dry weight. Biotests were carried out as described previously (Erban et al. 2016b) by adding 10 unsexed adults to the diet, with 12 replicates per bacterial strain and concentration. The control was the HDMd mite rearing diet. Mites were counted after 28 days using a dissecting microscope. The number of mites was used as the dependent variable, while the bacterial strain, control, and concentration were used as factors. Statistical analyses were performed in R v.4.1.2 (R Development Core Team 2021). We used ANOVA [WRS2 package (Mair and Wilcox 2020)] to compare the effect of diets and their interactions on the final density of mites.

### Sample processing

The transcriptome and genome samples were prepared as described previously (Hubert et al. 2023). All samples were homogenized for 30 s in a glass tissue grinder (Kavalier glass, Prague, Czechia) in 500 μL of lysis buffer on ice. A NucleoSpin RNA kit (Macherey–Nagel, Duren, Germany) was used for RNA extraction, with the following modifications: homogenized samples were centrifuged at 2,000×*g* for 3 s, and DNA was degraded by DNase I at 37 °C according to the manufacturer’s protocol (Riboclear plus, GeneAll, Lisbon, Portugal). RNA quality was evaluated using a NanoDrop instrument (NanoDrop One; Thermo Scientific, Waltham, MA, USA) and an Agilent 2100 Bioanalyzer (Agilent Technologies, Santa Clara, CA, USA). Samples were transported on dry ice to the MrDNA laboratory (Shallowater, TX, USA) for downstream processing and sequencing. DNA was extracted from the homogenates after overnight incubation with 20 μL of proteinase K at 56 °C using the QIAamp DNA Micro Kit (Qiagen, Hilden, Germany, cat. no. 56304) and following the manufacturer’s protocol for tissue samples. The concentration of the extracted DNA samples was quantified using a Qubit® dsDNA HS Assay Kit (Life Technologies), and the quality of the DNA was determined using a NanoDrop 2000 instrument. The average size of gDNA was determined using an E-Gel SizeSelect 2% Agarose Gel (Invitrogen) with a 1 kb ladder. The samples were sheared using a Covaris G-tube (Covaris). The average size of the sheared DNA was determined using a TapeStation 4200 system (Agilent Technologies). The samples were transported to the MrDNA laboratory in the same way as described above; samples for barcode sequencing were shipped to the University of Illinois in Chicago.

### Genome and transcriptome sequencing

Genome and transcriptome sequencing was performed in the MrDNA laboratory according to a protocol described previously (Hubert et al. 2023). For Illumina DNA sequencing, the libraries were prepared using a Nextera DNA Flex library preparation kit (Illumina) and subjected to paired-end sequencing for 500 cycles using a NovaSeq 6000 system (Illumina). For PacBio sequencing, the library was prepared with the SMRTbell Express Template Prep Kit 2.0 (Pacific Biosciences) and sequenced on a PacBio Sequel system (Pacific Biosciences). The SMRT Link Circular Consensus Sequencing workflow (SMRT Link v.9.0.0, CCS) was used to combine multiple subreads from the same molecule to generate a highly accurate consensus sequence. The samples were deposited in GenBank as project PRJNA625856. For transcriptomic analysis, poly-A selection and library preparation were performed by using KAPA mRNA HyperPrep Kits (Roche), and paired-end sequencing was performed for 500 cycles using a NovaSeq 6000 system (Illumina). The samples were deposited in GenBank as project PRJNA599071 (see Supplementary dataset—Tables S1 and S2 for *B*. *tropicalis* cDNA and proteins).

Read processing and genome and transcriptome assembly and annotation were performed as described previously (Hubert et al. 2023). Briefly, Illumina reads were trimmed with Trim Galore (Krueger 2021), corrected with fastQC (Andrews 2019) and then aligned together with the PacBio reads in hybrid SPADES v 3.14 (Antipov et al. 2016; Bankevich et al. 2012) for DNA-based reads and rnaSPADES (Bankevich et al. 2012). Bacterial sequences were annotated by Prokka (Seemann 2014), and predicted proteins were identified by KEGG using GhostKoala (Kanehisa et al. 2016). Reads were mapped onto two reference datasets using Bowtie2 (Langmead and Salzberg 2012; Langmead et al. 2009) and Minimap2 (Li 2018) for long sequences. Our bacterial reference dataset contained 16 genomes: *Buchnera*, *Mixta*, *Erwinia*, *Wigglesworthia*, *Pantoea agglomerans*, *Ca.* Pantoea carbekii, A-F bacterial symbionts of *Plautia stali*, *Tatumella citrea*, *Tamutella* sp., and the bacterial symbiont BFo1 of *Frankiella occidentalis*. The mite transcriptome reference dataset contained eight transcriptomes (*Tetranychus urticae*, *Metaseiulus occidentalis*, *Dermatophagoides pteronyssinus*, *Varroa destructor*, *Varroa jacobsoni*, *Ixodes scapularis*, *Rhipicephalus sanguineus*, and *Tribolium castaneum*). The mapped reads were assembled using Spades and Trinity (Grabherr et al. 2011). The assembled genome was then uploaded to the Type (Strain) Genome Server (TYGS) (see *Mixta mediterraneensis* determination, below). We identified the genome as *M. mediterraneensis*. We then used the original genome of *Mixta* (*Erwinia*) *mediterraneensis* (Boxberger et al. 2021) as a database and mapped the reads again in the same way as above. The reassembled genome and transcriptome were improved using Pilon (Walker et al. 2014). Then, they were reannotated using DFAST (Tanizawa et al. 2016, 2018) and KEGG mapper (Kanehisa and Goto 2000). The expression analyses of the *B. tropicalis* transcriptome were performed in CLC Workbench 22 (Qiagen, Venlo, The Netherlands) according to the recommended protocol (CLCbio 2023). We used the total number of reads as an expression parameter. The total number of reads per sample ranged from 12.4 to 16.7 × 10^6^ reads/sample. We standardized the data to the sample with the lowest number of reads (12.4 × 10^6^ reads).

### *Mixta mediterraneensis* determination

The genome sequence data were uploaded to the Type (Strain) Genome Server (TYGS), a free bioinformatics platform available at https://tygs.dsmz.de, for a whole genome-based taxonomic analysis (Meier-Kolthoff and Goker 2019). The analysis also made use of recently introduced methodological updates and features (Meier-Kolthoff et al. 2022). Information on nomenclature, synonymy and associated taxonomic literature was provided by TYGS’s sister database, the List of Prokaryotic names with Standing in Nomenclature (LPSN, available at https://lpsn.dsmz.de) (Meier-Kolthoff et al. 2022). The results were provided by TYGS on 2023-06-14. Determination of the closest type strain genomes was performed in two complementary ways; genomes were compared against all type strain genomes available in the TYGS database via the MASH algorithm, a fast approximation of intergenomic relatedness (Ondov et al. 2016), and the ten type strains with the smallest MASH distances chosen per user genome. An additional set of ten closely related type strains was determined via the 16S rDNA gene sequences. These were extracted from the user genomes using RNAmmer (Lagesen et al. 2007), and each sequence was subsequently BLASTed (Camacho et al. 2009) against the 16S rDNA gene sequence of each of the currently 19,121 type strains available in the TYGS database. This was used as a proxy to find the best 50 matching type strains (according to the bitscore) for each user genome and to subsequently calculate precise distances using the Genome BLAST Distance Phylogeny approach (GBDP) under the algorithm ‘coverage’ and distance formula d5 (Meier-Kolthoff et al. 2013). These distances were finally used to determine the 10 closest type strain genomes for each of the user genomes. For the phylogenomic inference, all pairwise comparisons among the set of genomes were conducted using GBDP, and accurate intergenomic distances were inferred under the algorithm ‘trimming’ and distance formula d5 (Meier-Kolthoff et al. 2013). One hundred distance replicates were calculated each. Digital DDH values and confidence intervals were calculated using the recommended settings of GGDC 3.0 (Meier-Kolthoff et al. 2013, 2022).

The resulting intergenomic distances were used to infer a balanced minimum evolutionary tree with branch support via FASTME 2.1.6.1 including SPR postprocessing (Lefort et al. 2015). Branch support was inferred from 100 pseudobootstrap replicates each. The trees were rooted at the midpoint (Farris 1972) and visualized with PhyD3 (Kreft et al. 2017). Type-based species clustering using a 70% dDDH radius around each of the 18 type strains was performed as previously described (Meier-Kolthoff and Goker 2019). Subspecies clustering was performed using a 79% dDDH threshold as previously introduced (Meier-Kolthoff et al. 2014).

### Barcode sequencing of microbial profiles

The bacteria and fungi in the mite microbiomes were characterized by barcode sequencing. The sequencing of bacteria was based on the V4 domain of the 16S rRNA gene (CS1_515F and CS2_806R primers) and fungi by ITS (primers ITS1f and ITS2) (Caporaso et al. 2012) according to protocols described previously (EMP 2022) at the DNA Services Facility of the Research Resources Center at the University of Illinois (Chicago, IL, USA) on the MiSeq platform (Illumina, San Diego, CA, USA) (Earley et al. 2015; EMP 2022). The sequences were demultiplexed, and the barcodes and primers were removed by the company. The forward and reverse sequences were aligned and processed with MOTHUR 1.47.0 (Schloss et al. 2009), according to the standard operating procedure (MiSeq SOP (Kozich et al. 2013)) and with the UPARSE 11 pipeline including UNOISE algorithms (Edgar 2013, 2016b) using a protocol that combined both programs (Sarikhani et al. 2017). Operational taxonomic units (OTUs) were classified with SINTAX commands (Edgar 2016a) in UPARSE using the training sets (Edgar 2022) obtained from the Ribosomal Database Project (Cole et al. 2014). The representative sequences of each OTU were then compared to those available in GenBank using BLASTn (Altschul et al. 1990). Raw DNA sequences were deposited under NCBI SRA: PRJNA916635. The bacterial and fungal OTUs with total abundances greater than 500 reads were used for standardized datasets, while the remaining sequences with total proportions lower than 2.3 and 2.4% of reads for bacteria and fungi, respectively, were discarded. The samples were deposited in GenBank under PRJNA916635 (Supplementary dataset—Table S1).

### qPCR

Amplification was carried out in a StepOnePlus™ Real-Time PCR System (Life Technologies, Grand Island, NY, USA) in 96-well plates using Luna qPCR Master Mix (New England Biolabs, Ipswich, MA, US). SYBR Green (Bio-Rad Laboratories, Veenendaal, The Netherlands) was employed as a double-stranded DNA (dsDNA) binding dye. We applied a routinely used protocol for standard preparation and qPCR detection (Kopecky et al. 2014). The qPCR standard was prepared from a cloned 16S rRNA gene amplicon (pGEM®-T Easy Vector, Promega) derived from PCR amplification of mite metagenomic DNA with ArsF 3′-GGGTTGTAAAGTACTTTCAGTCGT-5′ and ArsR2 3′-GTAGCCCTRCTCGTAAGGGCC-5′ provided an 803 bp fragment of 16S DNA. The competent bacterial cells with plasmids were inoculated in LB medium (Himedia, Mumbai, India) with 0.1 g/L ampicillin (cat. no. A01104.0005, Duchefa Biochemie, Haarlem, The Netherlands) for 16 h at 37 °C. The plasmid was then purified with a Wizard Plus SV Minipreps DNA purification system (cat. no. A1330, Promega) according to the manufacturer’s protocol. Plasmids were linearized by SacI restriction (cat. no. R6061, Promega) and cleaned with a Wizard SV gel and PCR Clean-Up system (cat. no. A9285). The concentration of the cleaned product was measured on a P330 Implen NanoPhotometer (Munich, Germany) and adjusted to 10 ng of DNA for each reaction. The primers Pant_F3 3′-GGAGGGTGCAAGCGTTAATC-5′ and Pant_R 3′-GAGACTCAAGCCTGCCAGTT-5′ were designed from 16S DNA of the bacterial symbiont, and PCR produced 124 bp. The qPCR conditions included hot start activation for 60 s at 95 °C, followed by 40 cycles of denaturation for 15 s at 95 °C, annealing for 30 s at 60 °C, melting for 15 s at 95 °C, 60 °C for 30 s and data collection after 0.5 °C up to 95 °C. The samples included 24 individual mites processed with surface sterilization according to a protocol described previously (Hubert et al. 2021) and feces extracts from 15 rearing chambers processed as described above. The resulting numbers were recalculated per mite and per chamber and transformed by log10.

### Protein analyses

Proteins for label-free proteomic mass spectrometry analysis were processed and further analyzed using a nanoLC‒MS/MS system employing an Orbitrap Fusion Tribrid mass spectrometer (Thermo) as previously described (Erban et al. 2020b, 2021; Hubert et al. 2023). Data were evaluated in MaxQuant v.2.2.0.0 using label-free quantification (LFQ) algorithms (Cox et al. 2014; Cox and Mann 2008) and the Andromeda search engine (Cox et al. 2011). The databases and search criteria were as described previously (Erban et al. 2016b, 2017; Erban and Hubert 2015). The protein data were processed in Perseus v.2.0.7.0. (Tyanova et al. 2016) and positive matches identified via data evaluation were utilized. For selected proteins, we ran manual searches in GenBank using pBLAST (Altschul et al. 1990) and applied HMMER (Eddy 1995) using the HMMER-Web server (Finn et al. 2011). Selected protein sequences were aligned using the T-COFFEE server (Notredame et al. 2000), followed by additional alignments in CLUSTAL v.2.1 (Larkin et al. 2007). Signal peptides were identified using SignalP v.6.0 (Teufel et al. 2022).

## Results

### Microbiome of *Blomia tropicalis*

**Fig. 1:**
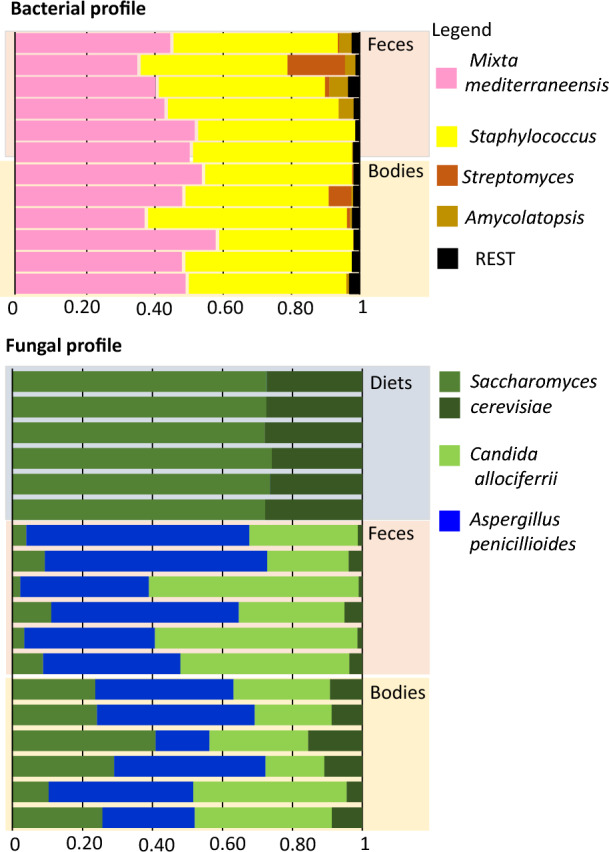
Bacterial and fungal profiles obtained from barcode sequencing of the V4 16S rRNA or ITS fragment from *Blomia tropicalis* bodies, feces and rearing diets. *Saccharomyces cerevisiae* formed two OTUs

Based on 16S rRNA data, the microbiome compositions in the mite body and feces were very similar. There were two dominant OTUs, classified as *M. mediterraneensis* and *Staphylococcus*, each of which contributed 40 to 50% of the reads in both niches (Fig. 1). The low-abundance OTUs were Actinomycetales (Streptomycetaceae and Pseudonocardiaceae) (Supplementary dataset—Table S3). The top three fungal species in the mite body and feces were *Saccharomyces cerevisiae*, *Aspergillus penicillioides*, and *Candida allociferi*. The fungal microbiome was influenced by the addition of *S. cerevisiae* to the mite rearing diet, and *S. cerevisiae* accounted for 100% of the reads in the diet samples. Previous experiments showed that mites use yeasts as a food source (Nesvorna et al. 2021).

**Fig. 2:**
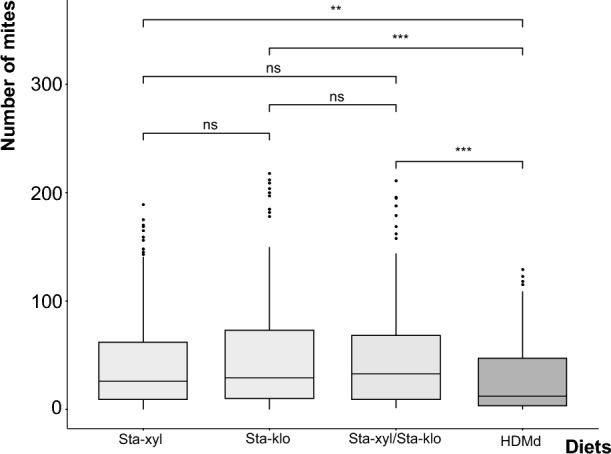
The effect of adding *Staphylococcus kloosii* (Sta-klo) and *Staphylococcus xylosus* (Sta-xyl) to the diet (HDMd) on the population growth of *Blomia tropicali*s in feeding experiments. The boxplots show the numbers of mites obtained after different treatments, and the lines indicate Tukey’s HSD. We did not find any differences among the concentrations of bacteria in the diet, so the concentrations were combined

*Staphylococcus* colonies were isolated from mite feces by inoculation in nutrient agar plates. The isolates were identified as *Staphylococcus kloosii* and *Staphylococcus xylosus* using 16S DNA (GenBank accessions: KY865751, KY865752) and rpoB markers. No other microorganisms were isolated by the plating method after several repeated attempts. *Staphylococcus* addition to the mite rearing diet (HDMd) at different concentrations and ratios influenced *B. tropicalis* population growth (measured as final population density) in comparison to the control diet without bacteria (Fig. 2) (F2,734 = 6549, P < 0.001). The strongest effect was shown by *S. kloosii*, with the mite population increasing twofold compared to that in the group fed the control (stored product) diet (Supplementary dataset—Table S4 for statistical analyses). Based on our 16S rRNA sequencing results, we did not find substantial amounts of bacterial reads in the pasteurized diet.

### *Mixta mediterraneensis*, a symbiont of *Blomia tropicalis*

Our genomic assembly (GenBank JASUAZ000000000) had 114 contigs (3.7 Mb, N_50_ = 31,524, GC = 51.76%, coverage = 27), with a BUSCO completeness of 100% using the Enterobacteriales database (Manni et al. 2021a, b). The genome contains 3,522 predicted proteins; of these, 2,522 proteins were assigned to KEGG pathways (Kanehisa et al. 2006). Our predicted 16S rRNA gene sequence returned a 100% match with the V4 16S rDNA fragment (see above). The mean numbers of copies were as follows: 10^6^ (range from 10^3^ to 10^7^) copies per mite; mite feces: 10^10^ (ranged 10^9^ to 10^10^) copies per chamber.

Based on a fastANI (MASH clustering based on average nucleotide identity; Jain et al. 2018), it had a 99.1% (1182 from 1201 fragments) similarity to *M. mediterraneensis* using the DFAST server (Tanizawa et al. 2016, 2018). This was confirmed by identification using Type (Strain) Genome Server (TYGS) (Meier-Kolthoff and Goker 2019, 2022). Both 16S DNA and genome comparisons classify it as the species *M. mediterraneensis* (Figs. S1, S2).

A KEGG analysis of *M. mediterraneensis* proteins (Supplementary dataset—Table S5) revealed 233 pathways and 84 complete modules (Table 1). The comparison of predicted KEGG proteins showed that the difference between the *B. tropicalis* symbiont and *M. mediterraneensis* strain Marseille-Q2057T was 9% of proteins. Among the compared KEGG proteins, 4% were unique to the *B. tropicalis* symbiont, while 5% of the KEGG proteins were unique to the *M. mediterraneensis* strain Marseille-Q2057T (Fig. S3). The KEGG modules included those corresponding to essential amino acid production, which are present in most *Pantoea* species but absent in *Ca.* P. carbekii (Kenyon et al. 2015).

**Table 1:** Comparison of the predicted *Mixta mediterraneensis* genome, the *Blomia tropicalis* transcriptome and the bacterial isolate Marseille-Q2057^T^. The transcriptome was compared to a previously published *B. tropicalis* strain (Xiong et al. 2022). Numbers of predicted (p.) genes as KEGG included duplicates. The comparisons are based on the Ghost Koala annotation (Kanehisa et al. 2016)

Gen/trans	No. p. genes	No. p. genes as KEGG	No. KEGG	Pathways	KEGG_modules
*Blomia*_trans	18,164	9661	5750	438	68
(expression)	17,082	9306	5670	433	68
Chinese_*Blomia*	16,590	7811	5310	430	54
*Mixta mediterraneensis*	3522	2523	2464	233	84
*M.* *mediterraneensis* Marseille-Q2057^T^	4064	2550	2227	239	80
mite + bact				444	126

Our shotgun bottom-up proteomic analysis enabled the identification of 136 *M. mediterraneensis* proteins (Supplementary dataset Table S5) in both the mite body and feces (Figs. S4, S5). However, protein presence/absence and abundance were significantly different in these two niches (ANOSIM: Jaccard index; R = 0.5074; P = 0.0045; Bray‒Curtis; R = 0.5093; P < 0.0037). Comparing both analyses, the differences were caused by protein absence/presence (Fig. S5) but not protein abundance, indicating different protein production of symbionts in mite bodies and feces. A high number of proteome-identified proteins were involved as transporters, including phosphate, lysine/arginine/ornithine, histidine, arginine, glutamate/aspartate, cystine, branched amino acids, D-methionine and urea transporters. The metallics transporters included Fe-siderophores, Fe(III) hydroxamate, and zinc. The vitamin transporters included vitamin B12; lipoprotein and heme transporters were found.

### Transcriptomic and proteomic analyses identified extracellular digestive enzymes

The *B. tropicalis* genome (JACEGP000000000) (Hubert et al. 2023) had 7,171 contigs (31 Mb, N_50_ = 5,996 bp), 18,164 genes and 5,750 KEGG pathways (Supplementary dataset Tables S2 and S7). A BUSCO analysis with the Arachnida database estimated its completeness to be 95%, with 9.1% gene duplication. In contrast, a previous transcriptome had 16,590/14,899 predicted genes and 5,310 KEGG pathways (Xiong et al. 2020, 2022). We identified 433 KEGG pathways and 68 modules (Table 1) in the transcriptome-level analyses. The *B. tropicalis* transcriptome had 51 predicted enzymes with hydrolytic activity, among which 40 were identified in protein profiles. Among the identified enzymes, 20 were present in both the mite body and feces (Supplementary dataset Tables S7 and S8).

The enzymes with the highest levels in the protein profile (ranked from highest to lowest) were alpha amylase (JGLJHAHI_01112), triacylglycerol lipases (JGLJHAHI_03418 and JGLJHAHI_03527), leucyl aminopeptidase (JGLJHAHI_10398), carboxypeptidase Q (JGLJHAHI_09345), hexosaminidase (JGLJHAHI_13622) and chitinases (JGLJHAHI_17563 and JGLJHAHI_08029). The high levels of both chitinases in the feces indicated that they are expressed in the gut lumen and do not participate in cuticle formation. All these enzymes contained signal peptides, indicating extracellular functions. Leucyl aminopeptidase, however, did not have a signal peptide, but PHMMER identification revealed a transmembrane region. Leucyl aminopeptidase activity was found in the *B. tropicalis* culture medium, supporting its presence in feces. Cathepsin D (JGLJHAHI_07681) has been identified in *B. tropicalis* and many other astigmatid mites (Fig. S7) (Supplementary dataset Table S9).

We found 7 clusters of predicted genes encoding chitinase activity (Fig. S8) in the *B. tropicalis* transcriptome (Supplementary dataset Tables S10, S11). Among these chitinases, JGLJHAHI_05682 was novel, i.e., not discovered previously in *B. tropicalis*, while glycosyl hydrolase 18 (KAH9407551, KAI2807268 and JGLJHAHI_02728) was unique to *B. tropicalis* (absent in other mites) (Fig. S9). Structural alignment with chitinases with known hydrolytic activity toward bacterial cell walls (lysosomal) (de Medeiros et al. 2018) (i.e., *Aspergillus terreus*: XP_001214802, *Pseudomonas aeruginosa*: WP_058162064 and *Monascus purpureus*: TQB68084) (Figs. S9–S15, Supplementary dataset Table S12) identified JGLJHAHI_17563 (allergen group 15) as an enzyme with possible activity toward bacterial cell walls.

## Discussion

### *Staphylococcus* and *M. mediterraneensis* are the main microbes in *Blomia tropicalis*

The allergen-producing mite *B. tropicalis* hosts the symbiotic bacterium *M. mediterraneensis*. The bacterium is present in the mite body and feces and accounts for more than 50% of the mite microbiome. The remaining gut microbiome covered *Staphylococcus* growing in the mite culture. The pasteurized mite rearing diet was almost without bacterial reads, indicating that bacteria were associated with the mite rather than being introduced via the diet. Our analyses and biotest results suggest either that the mite *B. tropicalis* uses *Staphylococcus* as a food source or that these bacteria promote mite population growth indirectly. The presence of *M. mediterraneensis* in mite feces and its genome size and gene conservation all indicate that this bacterium is an extracellular symbiont. We suggest that *M. mediterraneensis* can interact with other members of the mite microbiome via invasin production and manipulation of *B. tropicalis* phagocytosis. The mite exhibited high levels of chitinases and cathepsin D in the proteome of the mite body, with the ability to hydrolyze bacterial cell walls, but it lacked lysozyme, which is provided by the bacterial symbiont *M. mediterraneensis*. The *M. mediterraneensis* genome indicated that the bacterium improves the nitrogen metabolism of its host.

### Genome predicted function of *M. mediterraneensis*

*Mixta mediterraneensis* can synthesize thiamin, riboflavin, pyridoxal-P, pantothenate, biotin and lipoic acid. The spectrum of vitamin pathways identified was the same as that of *P. stali* symbiont F (Hosokawa et al. 2016). The related bacterium *Ca.* Pantoea carbekii produces riboflavin, folate, and lipoic acid but not biotin (Kenyon et al. 2015). Our genomic data indicated that *M. mediterraneensis* reduces nitrate to nitrite (narG, narI) and vice versa (narG, narI, nasB); however, we did not confirm the reduction of nitrate to ammonia. In contrast, metabolic pathway analyses of the *P. stali* F symbiont (Hosokawa et al. 2016) showed the presence of enzymes for both dissimilatory and assimilatory nitrate reduction. *Mixta mediterraneensis* can utilize glutamine to produce amino acids. The presence of glnA, gltB, gltD and GLUD1_2 indicated the ability to convert ammonia to amino acids through glutamate and glutamine. The alternative pathway is nitrogen recycling from arginine through the arginine succinyl transferase pathway, which has been reported in our study and in *Ca.* P. carbekii (Kenyon et al. 2015). The three symbionts, *Ca.* P. carbekii, F of *P. stali* and *M. mediterraneensis*, have a complete sulfate-sulfur assimilation pathway. This pathway is well documented in free-living *P. agglomerans* (Shariati et al. 2017).

**Fig. 3:**
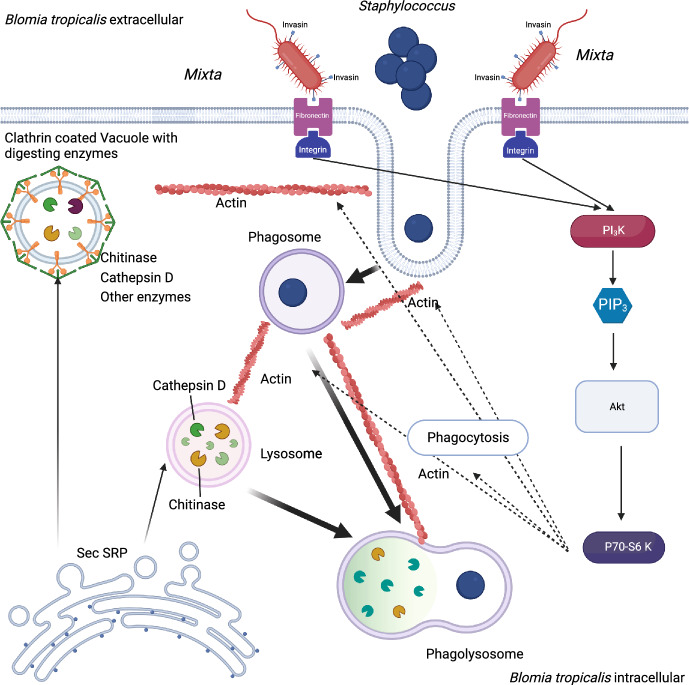
Schematic representation of potential interactions between *Mixta mediterraneensis* and *Staphylococcus* in the midgut of *B. tropicalis*. Endocytosis is mediated by *M. mediterraneensis* invasins, which act as mediators, and the PI3-Kinase/Akt signaling pathway. Activation starts with the binding of invasins to fibronectin (FN1)/integrin (CD29); the following cascade involves the activation of PIK3 and production of phosphatidylinositol-3,4,5-trisphosphate. This results in the activation of AKT (RAC serine/threonine-protein kinase [EC:2.7.11.1]) and RPS6KB phosphorylation. The phosphorylation of RPS6KB induces actin production and cell proliferation. Endocytosed *Staphylococcus* is degraded in lysozymes and phagosomes by cathepsins, chitinases, and subsequently lysosomal enzymes and predicted chitinases with hydrolytic activity toward bacterial cell walls. Moreover, starting with the Sec SRP system, there are some enzymes present in two types of vacuoles: lysosome precursors and precursors of secreting vacuoles (proven by the presence of cathepsin D and chitinases in both the body and feces)

Members of Enterobacteriaceae use a type III secretion system to manipulate the cytoskeletal machinery of host cells, including the modulation of lamellipodium formation and subsequent endocytic trapping in internal host cells (Koga et al. 2012). The type III system is complete in the F symbiont of *P. stali*, but it is absent in *Ca.* P. carbekii (Hosokawa et al. 2016) and our assembled genome of *M. mediterraneensis* with and without yscV. The Sec-SRP and twin arginine targeting pathways were complete. The other predicted proteins in our assembled genome of *M. mediterraneensis* are involved in secretion systems, including those of Type I (tolC), Type II (gspD, gspE, gspF gspG and gspO) and Type VI (icmF, DotU) proteins. We identified invasins yeeJ, sipD, and sipB, which are responsible for the initial step of infection of the host cell epithelium (Fig. 3). In *M. mediterraneensis*, we identified 33 proteins associated with biofilm formation, including proteins involved in flagellar regulation and assembly (e.g., flhC, fhlD, fliC) and the complete stewartan EPS biosynthesis pathway (expI, expR and rscA).

We identified all microcin C transporters (yejA-F). Microcin C is a compound used as a defense mechanism, and nonhydrolyzable aspartyl–adenylate is imported into bacteria when the carrier is removed by proteolytic processing to release a potent aspartyl tRNA synthetase inhibitor (Severinov and Nair 2012). However, we did not identify the MccB and MccA proteins as being part of the microcine C biosynthetic pathway (Severinov and Nair 2012).

### Intracellular digestion of *Staphylococcus* through lysosomes

We sought to identify how *Staphylococcus*, a gram-positive bacterium, might be utilized as a source of nutrients in the presence of gram-negative *M. mediterraneensis*. The digestion of bacterial cells has been suggested to be carried out by lysozyme in combination with cathepsin D (lysosomal aspartyl protease) (Terra and Ferreira 1994). However, lysozyme was present only in the genome of the mite symbiotic bacterium *M. mediterraneensis* (Supplementary dataset Table S4) and not in the mite genome. This suggests that *M. mediterraneensis* may cooperatively provide lysozyme to be used by mite hosts for various functions, including antimicrobial defense and digestion. Cathepsin D was highly expressed in the body of *B. tropicalis*, while in feces, the cathepsin D expression level was 10 times lower. We did identify its signal peptide as well as in chitinase, which contradicts the predicted intracellular digestion.

We suggest that *Staphylococcus* bacteria are phagocytosed (Fig. 3). Chitinase and cathepsin D can partially hydrolyze the bacterial cell walls in the gut lumen prior to phagocytosis. The next step of digestion occurs inside these phagocytosed vesicles through pathways including endocytosis (112 KEGG genes) and the lysosome (66 KEGG genes) and phagosome (48 KEGG genes) pathways (Supplementary dataset Table S10). The high expression and protein levels of cathepsins CTSL and CTSK, lysosomal enzymes LIPA, PSAP and LAMAN, and lysosomal membrane protein CD107 in the mite body profile were characteristic of the lysosome pathway (Supplementary dataset Table S10). Surprisingly, LAMAN (lysosomal alpha-mannosidase) and CTSK were highly expressed in the feces. The involvement of phagosomes was supported by high expression of ACTB_G1, which accelerates actin polymerization and phagocytic cap formation. With respect to endocytosis, we found high expression and protein profiles of HSPA1s (heat shock proteins) and CTLc (clathrin heavy chain). Phagosome formation is characterized by high expression and abundance of proteins involved in phagosome formation, such as actin (TUBA, TUBB) and CALR. Another highly expressed and abundant protein in phagosomes was cathepsin CTSL, which exerts hydrolytic activity toward protein fragments from bacterial cell walls. The low protein content of CTSL in feces indicated intracellular activity.

Endocytosis is suggested to be mediated by interactions with the PI3-kinase/Akt signaling pathway (Fig. 3) in *B. tropicalis*. We identified the complete PI3-kinase/Akt signaling pathway (Supplementary dataset Table S12). Activation starts with the binding of invasins to fibronectin (FN1)/integrin (CD29); the subsequent cascade involves the activation of PIK3 and production of phosphatidylinositol-3,4,5-trisphosphate. These findings suggested that *M. mediterraneensis* invasion sipB can act as a mediator and activate fibronectin (FN1)/integrin (CD29). This results in the activation of AKT (RAC serine/threonine-protein kinase [EC:2.7.11.1]) and RPS6KB phosphorylation. The phosphorylation of RPS6KB induces actin production and cell proliferation. Endocytosed *Staphylococcus* is degraded in lysozymes and phagosomes by cathepsins, chitinases, and subsequently lysosomal enzymes and predicted chitinases with hydrolytic activity toward bacterial cell walls (Fig. 3). Based on our data, we cannot distinguish whether this pathway is employed for bacterial utilization and/or represents an immune response.

Moreover, starting with the Sec SRP system, there are some enzymes present in two types of vacuoles, one is the lysosome precursor and the other is the precursor of the secreting vacuole, as proven by the presence of cathepsin D and chitinases in both the body and feces. The histological observation of the mite ventriculus and caeca (anterior midgut) showed high vacuole proliferation (Erban et al. 2016a; Smrz and Catska 1989), which supported our findings. In addition, the life cycle of anterior midgut cells is terminated by apoptosis, and their contents are emptied into the midgut lumen (Sobotnik et al. 2008). However, further experiments are needed to confirm the intracellular digestion of *Staphylococcus* by lysosomes. Additionally, the suggested immunological mechanisms should be experimentally confirmed in future studies.

### The suggested mite metabolic waste recycling by *M. mediterraneensis*

**Fig. 4:**
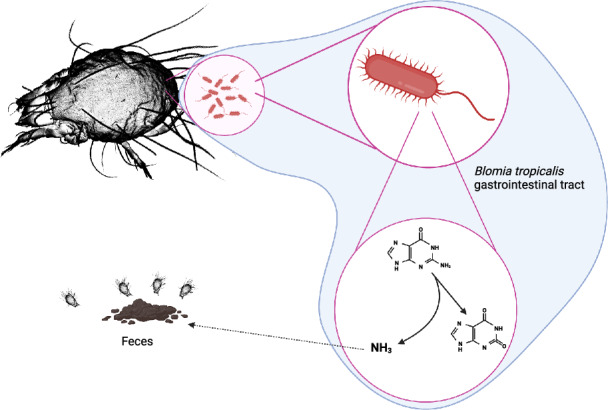
Schematic representation of potential interactions between *Mixta mediterraneensis* and *Blomia tropicalis* during nitrogen waste recycling. The bacterium converts guanine to hypoxanthine and degrades hypoxanthine to ammonium. Ammonium is known to function as a kairomone to attract mites to feces

The recycling of nitrogen waste metabolism is suggested as one of the benefits of bacterial symbionts to eliminate nitrogen hunger of their host living in niches with low amounts of nitrogen (Douglas 2009). The mites are known to infest various stored plant products (Hughes 1976), which should be poor in nitrogen, although they can switch to feeding on fungi and bacteria (Hubert et al. 2015). In contrast to uric acid (Douglas 2009) as a waste compound in insects, mites produce guanine as a nitrogen waste metabolic product (Levinson et al. 1991; McEnroe 1961). It is believed that guanine is deposited in crystal form as birefringent, conspicuously silver, and appears as concentric circular spherites in mite fat tissue (Smrz and Catska 2010). In *Tyrophagus putrescentiae*, extensive feeding on fungi resulted in massive accumulation of those spherites in mite fat tissues and damage to internal organs (co called white-body syndrome) (Smrz and Catska 1989). However, guanine is excreted in the feces and shows kairomone function in mites in *Acarus siro* (Levinson et al. 1991). It was experimentally proven that bacteria equipped with guanine deaminase (e.g., *Bacillus subtilis*) induced their growth on purines as the nitrogen source (Nygaard et al. 2000). The analyses of KEGG pathways of *M. mediterraneensis* revealed guaD (K01487 guanine deaminase [EC:3.5.4.3]) reducing guanine to xanthine and complete pathways to produce allantoin and its conversion to oxalate and ammonia (Fig. 4). Ammonium is experimentally documented to attract mites and stimulate their aggregation and feeding, as shown in experiments using *A. siro* (Levinson et al. 1991). In contrast to ammonium, guanine is an attractant (kairomone) for mites in a short concentration range (Levinson et al. 1991). There is a linear relationship between ammonium and its kairomone function in mites. This means that ammonia production from guanine did not change mite aggregation behavior in comparison to the situation described in *A. siro* (Levinson et al. 1991). The ammonium stimulation of aggregation and feeding on the feces containing *M. mediterraneensis* should be a mechanism of symbiont feces oral transmission to the gut of newly emerged mites feeding on the feces of their parents. Such a type of bacterial transition is well documented for cockroaches and termites (Nalepa et al. 2001; Wada-Katsumata et al. 2015). Bacterial cultivation and manipulative experiments are necessary to confirm metabolic waste recycling.

The gram-negative bacterium *M. mediterraneensis* previously isolated from human skin swabs was identified as gut symbiont of the mite *B. tropicalis*. We suggested a fecal–oral route of transmission. Genomic analysis revealed that symbionts can help their host by nitrogen recycling to convert mite nitrogenous waste guanine into ammonia. Moreover, ammonium has a kairomone function and is an attractant to mites to aggregate in their feces. The mite possesses digestive wall-degrading enzymes (chitinases and cathepsin D) to utilize gram-positive *Staphylococcus* bacteria growing in the mite feces or diet remnants. These interactions provide insight into the nutritional biology of this medically important mite.

### Supplementary Information

Below is the link to the electronic supplementary material.

Supplementary file1 (DOCX 7915 KB)

Supplementary file2 (XLSX 18341 KB)

## Data Availability

Sequences and samples obtained during this work have been submitted to the NCBI Respiratory database (SUB13263795, SUB7787303). The accession number for the raw nLC‒MS/MS runs reported in this paper is MassIVE MSV000091854 (10.25345/C5VD6PF6P) or PXD041972. Furthermore, we provide the entire “combined/txt” folder from MaxQuant data processing and the protein databases used for the search for download.
