# Supplementary material for: Mixta mediterraneensis as a novel and abundant gut symbiont of the allergen-producing domestic mite Blomia tropicalis
Source: Exp Appl Acarol. 2024 Jan 16;92(2):161–81. doi: 10.1007/s10493-023-00875-3 (PMC10927894; doi:10.1007/s10493-023-00875-3)
Supplement: Supplementary file 1 — Supplementary file1 (DOCX 7915 KB) [file 10493_2023_875_MOESM1_ESM.docx]

**Supplementary figures S1–S15**

**Title:** *Mixta mediterraneensis* as a novel and abundant gut symbiont of the allergen-producing domestic mite *Blomia tropicalis*

**Authors:** T. Erban, B. Sopko, P. B. Klimov, J. Hubert

**Journal:** Experimental and Applied Acarology

**Fig. S1** Tree inferred with FastME 2.1.6.1 (Lefort et al. 2015) from GBDP distances calculated from 16S rDNA gene sequences in Type (Strain) Genome Server (TYGS) (Meier-Kolthoff and Goker 2019; Meier-Kolthoff et al. 2022). The compared 16S DNA of the “genome” obtained from *Blomia tropicalis* clustered to *Mixta mediterraneensis*. The branch lengths are scaled in terms of the GBDP distance formula d5. The numbers above branches are GBDP pseudobootstrap support values > 60 % based on100 replicates, with an average branch support of 58.4 %. The tree was rooted at the midpoint (Farris 1972).


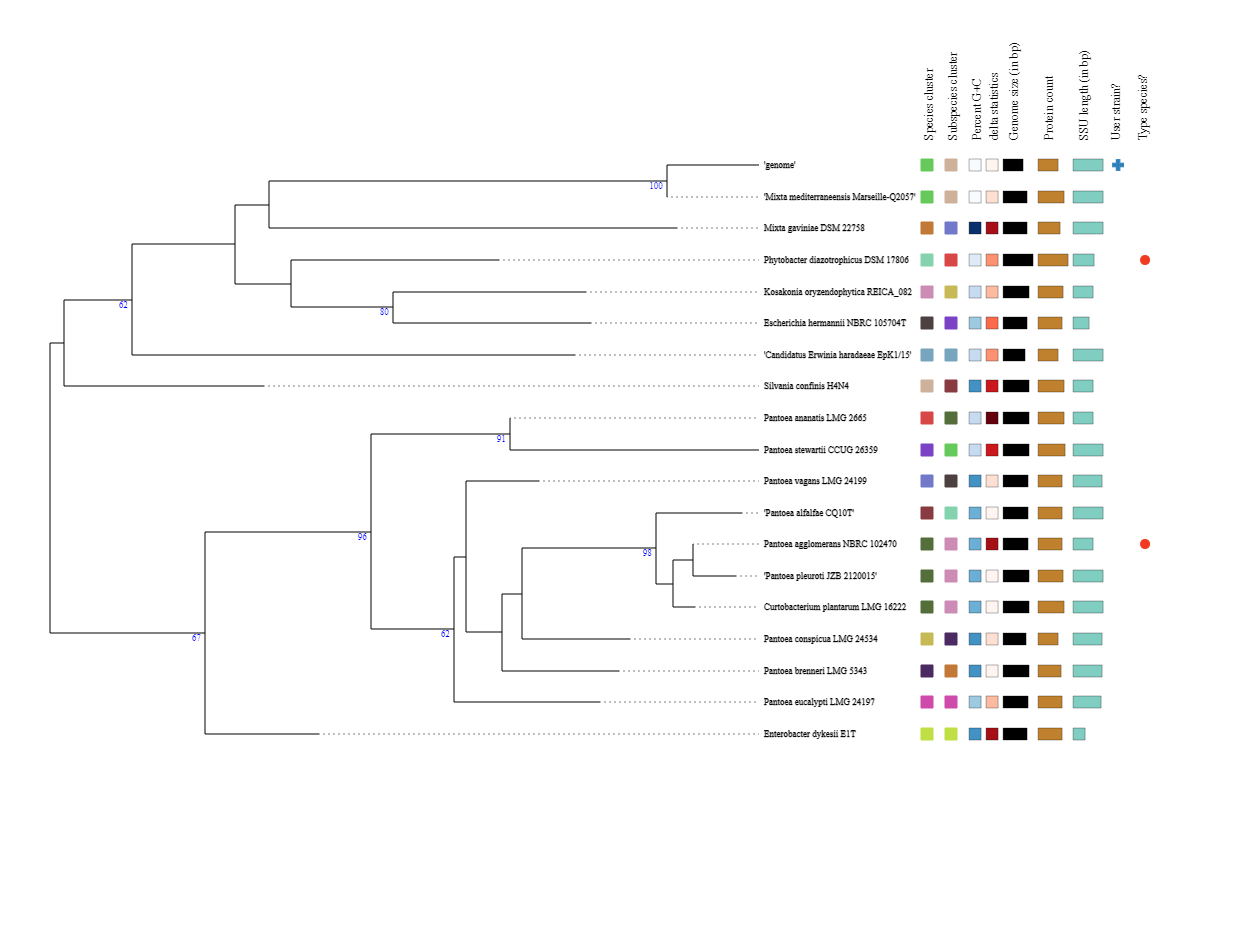


**Fig. S2** Tree inferred with FastME 2.1.6.1 (Lefort et al. 2015) from GBDP distances calculated from genome sequences. The compared “genome” obtained from *Blomia tropicalis* clustered with *Mixta mediterraneensis*. The branch lengths are scaled in terms of the GBDP distance formula *d_5_*. The numbers above branches are GBDP pseudobootstrap support values > 60 % based on 100 replicates, with an average branch support of 95.3 %. The tree was rooted at the midpoint (Farris 1972).**
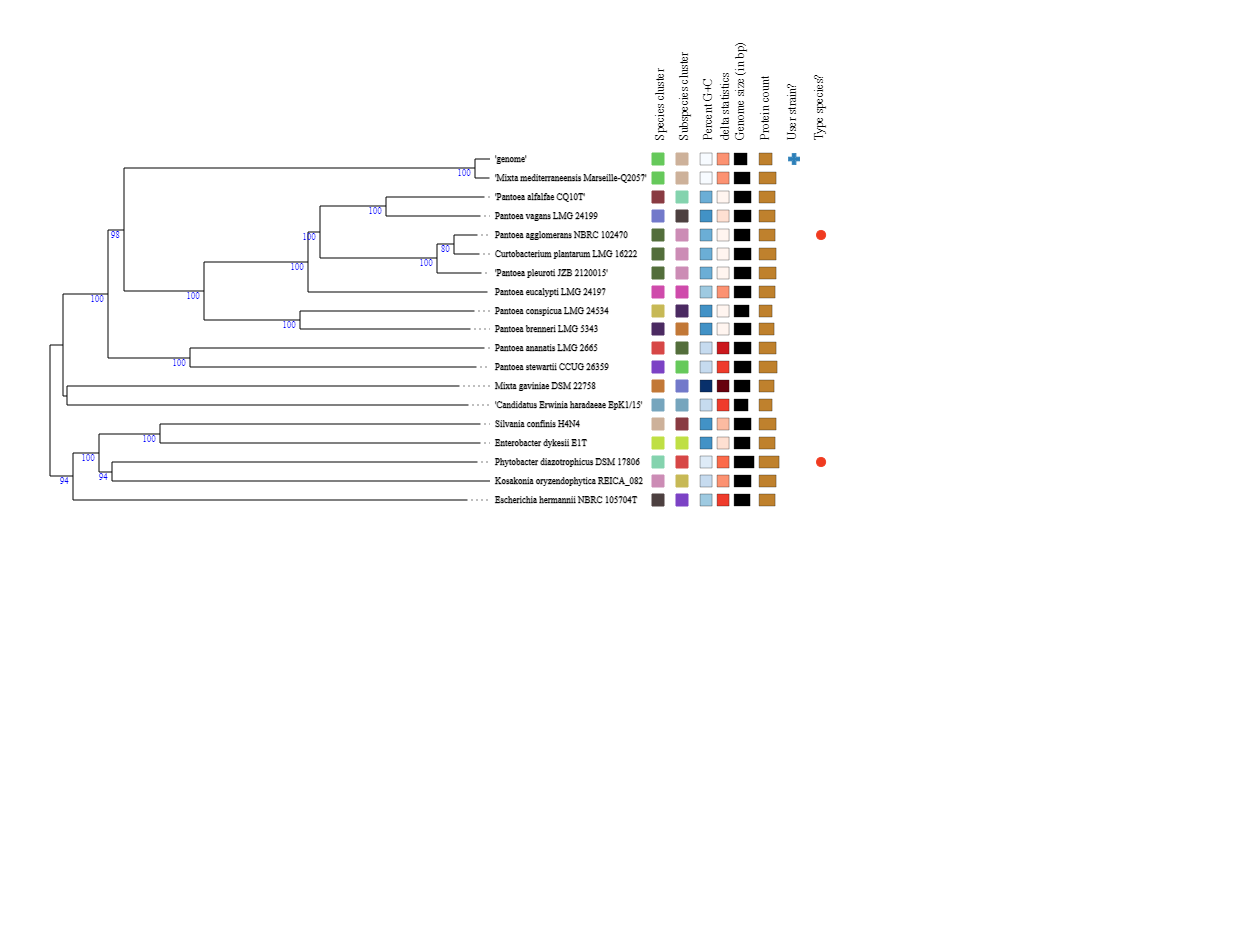
**

**Fig. S3** Venn diagram comparison of predicted KEGG proteins in the *Erwinia mediterraneensis* Marseille-Q2057^T^ strain and symbionts of *Blomia tropicalis*, *Pantoea carbeki* and the F_symbiont of *Plautia stali*.

**
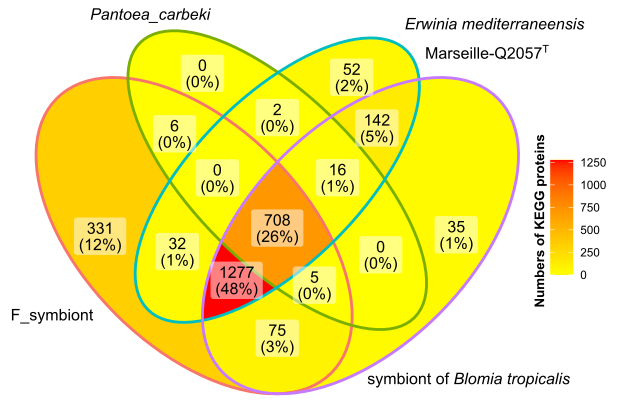
**

**Fig. S4** Histograms showing the proportion of *Mixta mediterraneensis* (red) in the proteomes of *Blomia tropicalis* (blue) mite bodies: A1-3 =1,000 individually collected adult mites, MA1-3 = pooled samples of mites in different stages of development, including eggs. LFQ = label-free quantitation based on precursor signal intensity.


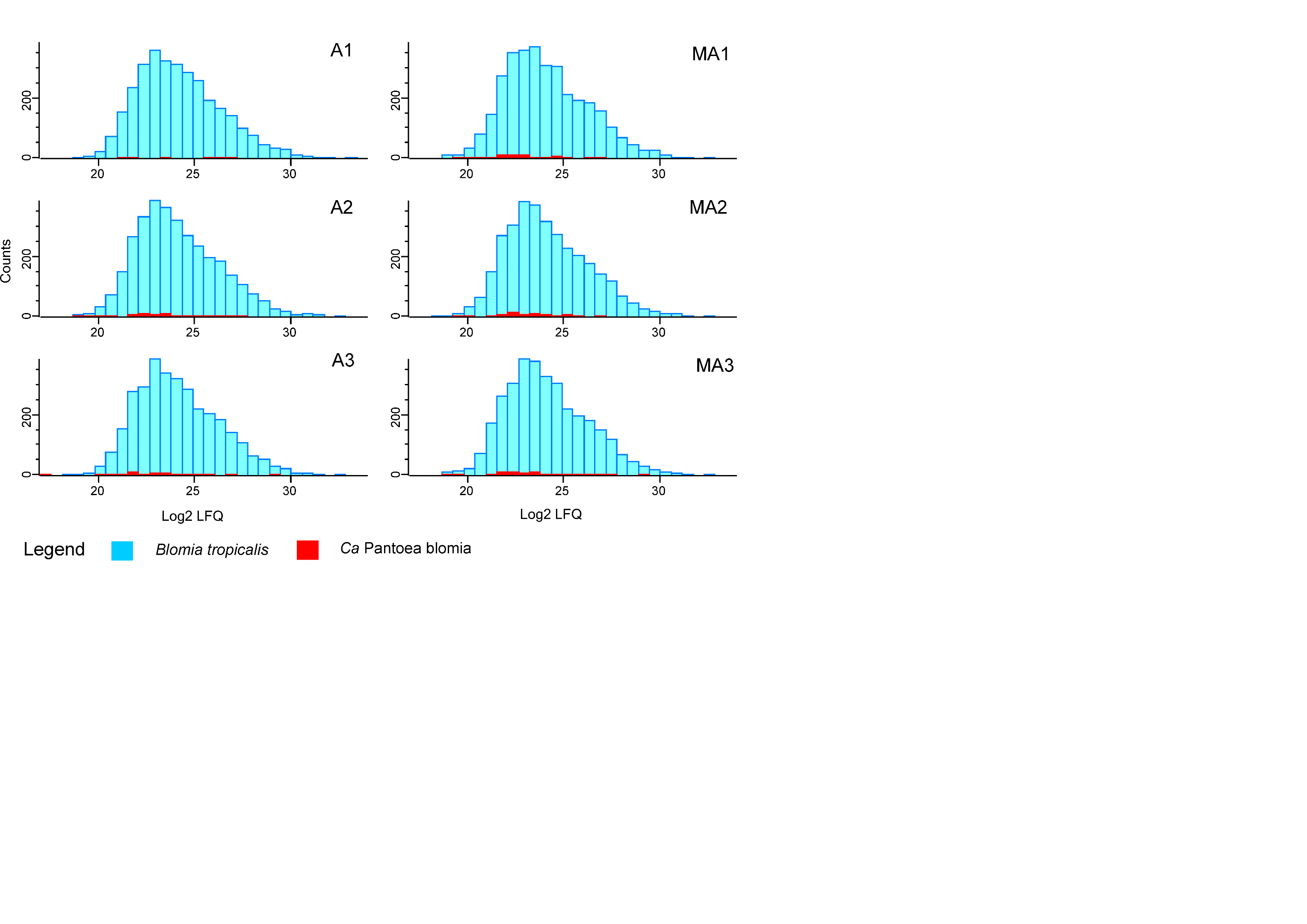


**Fig. S5** Histograms showing the proportion of *Mixta mediterraneensis* (red) in the proteomes of *Blomia tropicalis* (blue) mite feces. Samples E1-3 = water extracts of feces; FP = detergent-buffer extracts of the remaining pellet of the water extract. LFQ = label-free quantitation based on precursor signal intensity.**
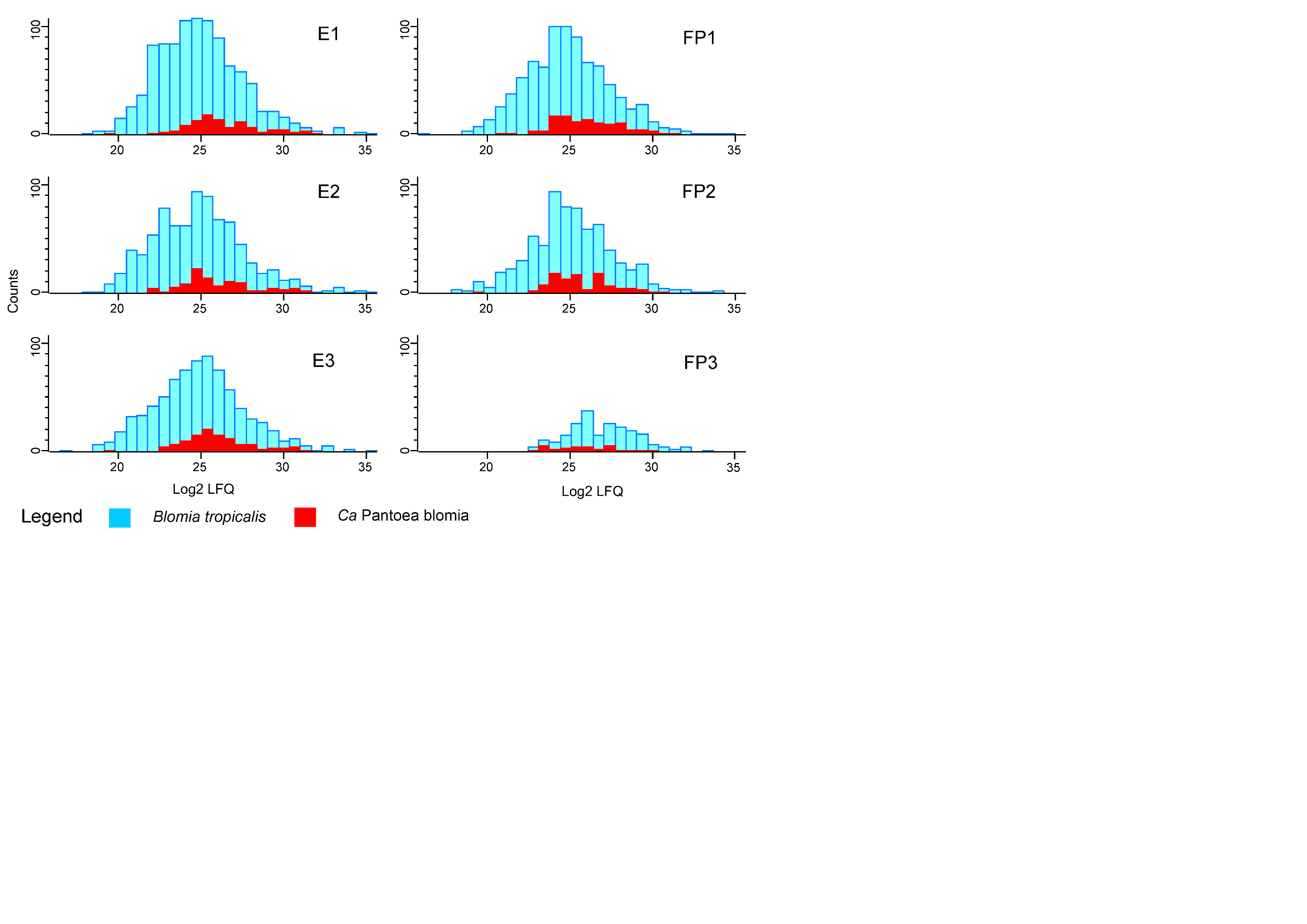
**

**Fig. S6** Comparison of identified genes of *Mixta mediterraneensis* in genomes and proteomes using all predicted proteins (**A**) and proteins assigned to KEGG (**B**). Venn diagrams were constructed.

**
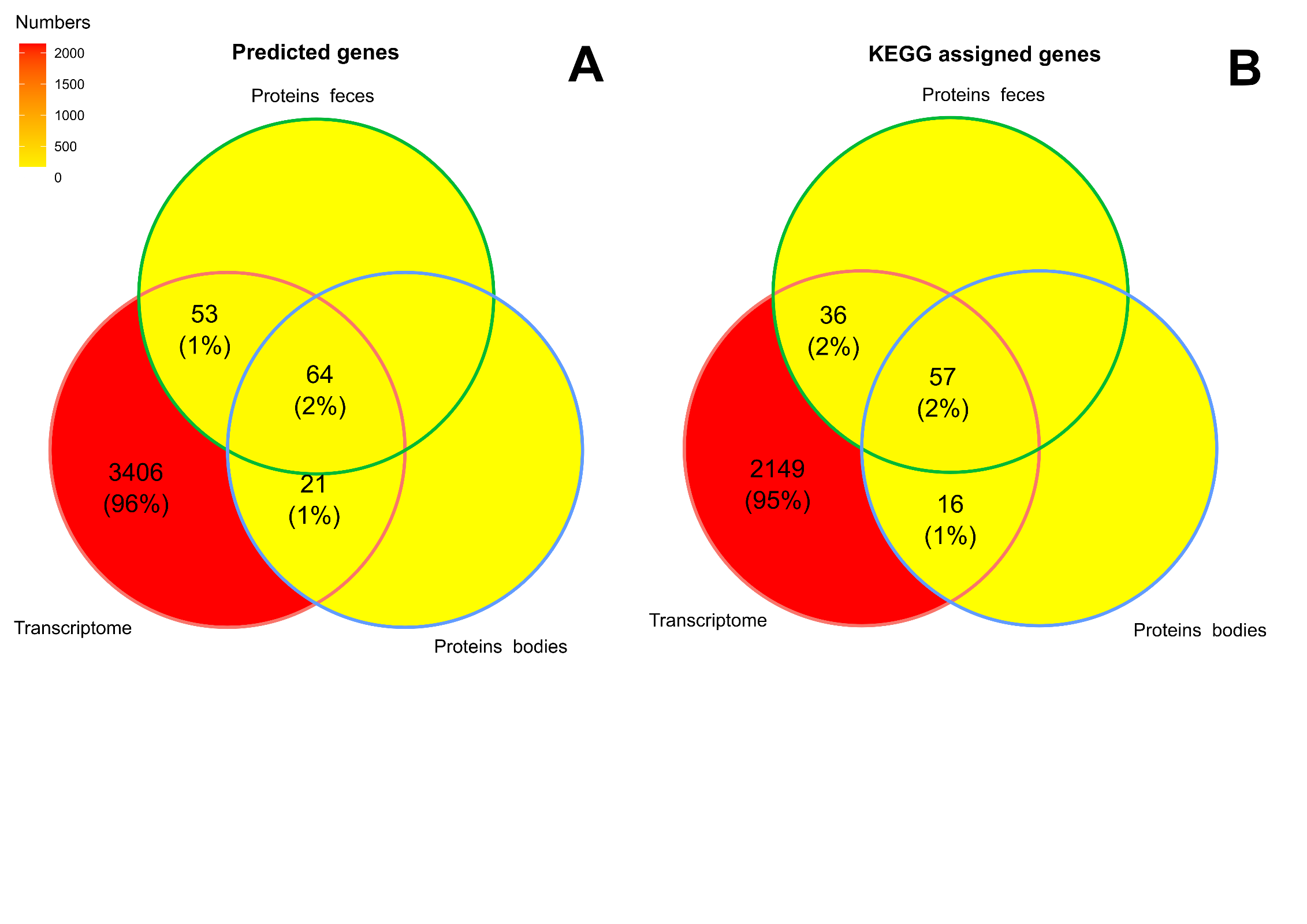
**

**Fig. S7** Identification of *Blomia tropicalis* cathepsin D (lysosomal aspartyl protease) based on homology with other mite proteins using GenBank data. Alignment was performed using T-Coffee, and the phylogenetic tree was inferred in PHYML. Our predicted *B. tropicalis* protein is shown in red, and GenBank *Blomia* proteins are shown in blue. Bootstrap support values are provided for each branch. All proteins are listed in Table S7. The outgroup sequence XP_017492495 originated from the *Rhagoletis zephyria* genome contaminated by *Tyrophagus putrescentiae* DNA (Hubert et al. 2019b).


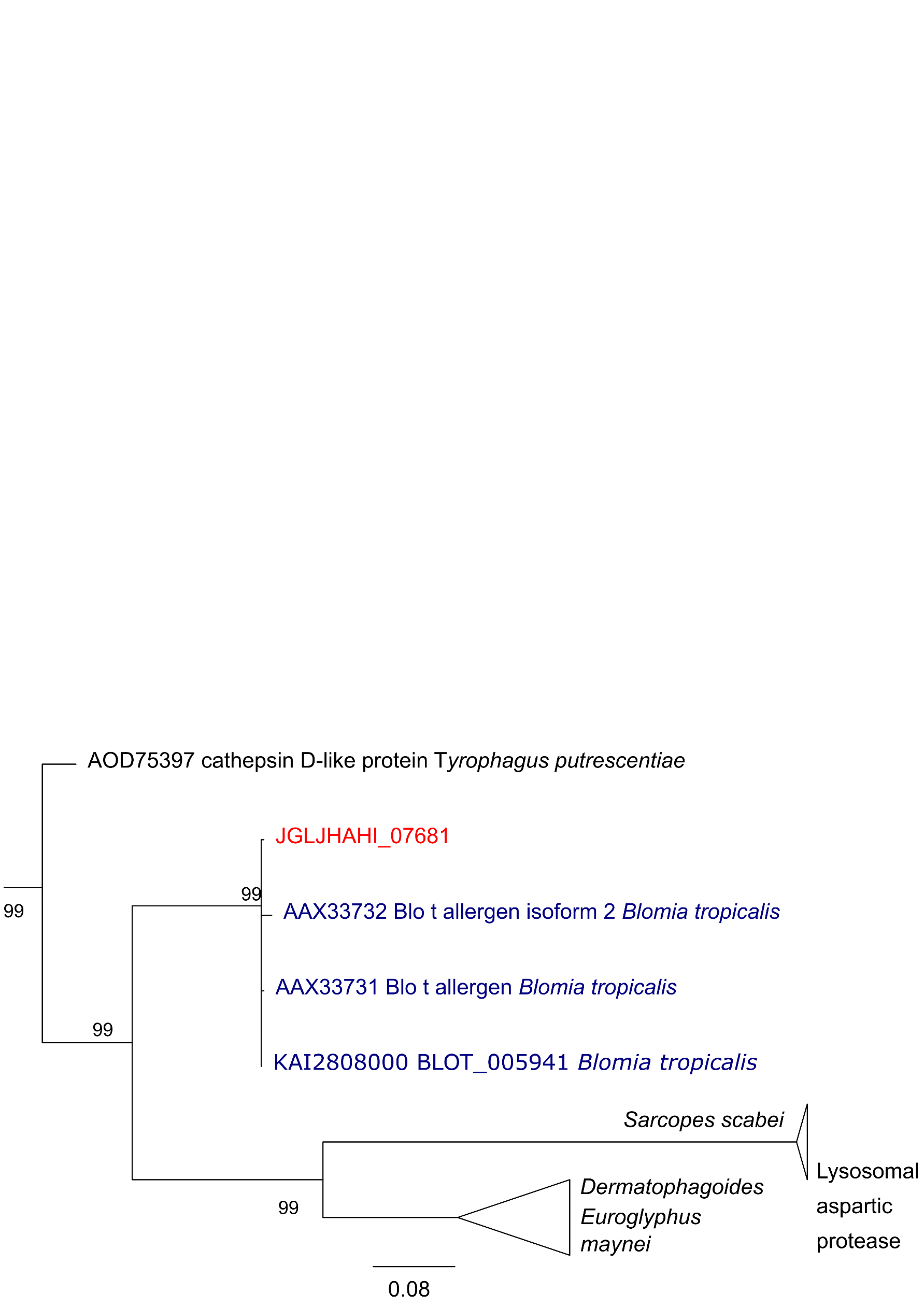


**Fig. S8** Identification of *Blomia tropicalis* chitinases using GenBank data. Our *B. tropicalis* proteins are shown in red font, and GenBank *B. tropicalis* proteins are shown in blue font. Different colors on the tree indicate seven types of chitinases: (i) chitinase-like, (ii) glycosyl hydrolase 18; (iii) chitinase 3-like; (iv) acidic mammalian-like chitinase; (v) Cht7/9; (vi) Sar S5 ; and (vii) allergen group 15. This tree was inferred as described in Fig. S6; all proteins are listed in Table S8. Bootstrap support values are provided for each branch.


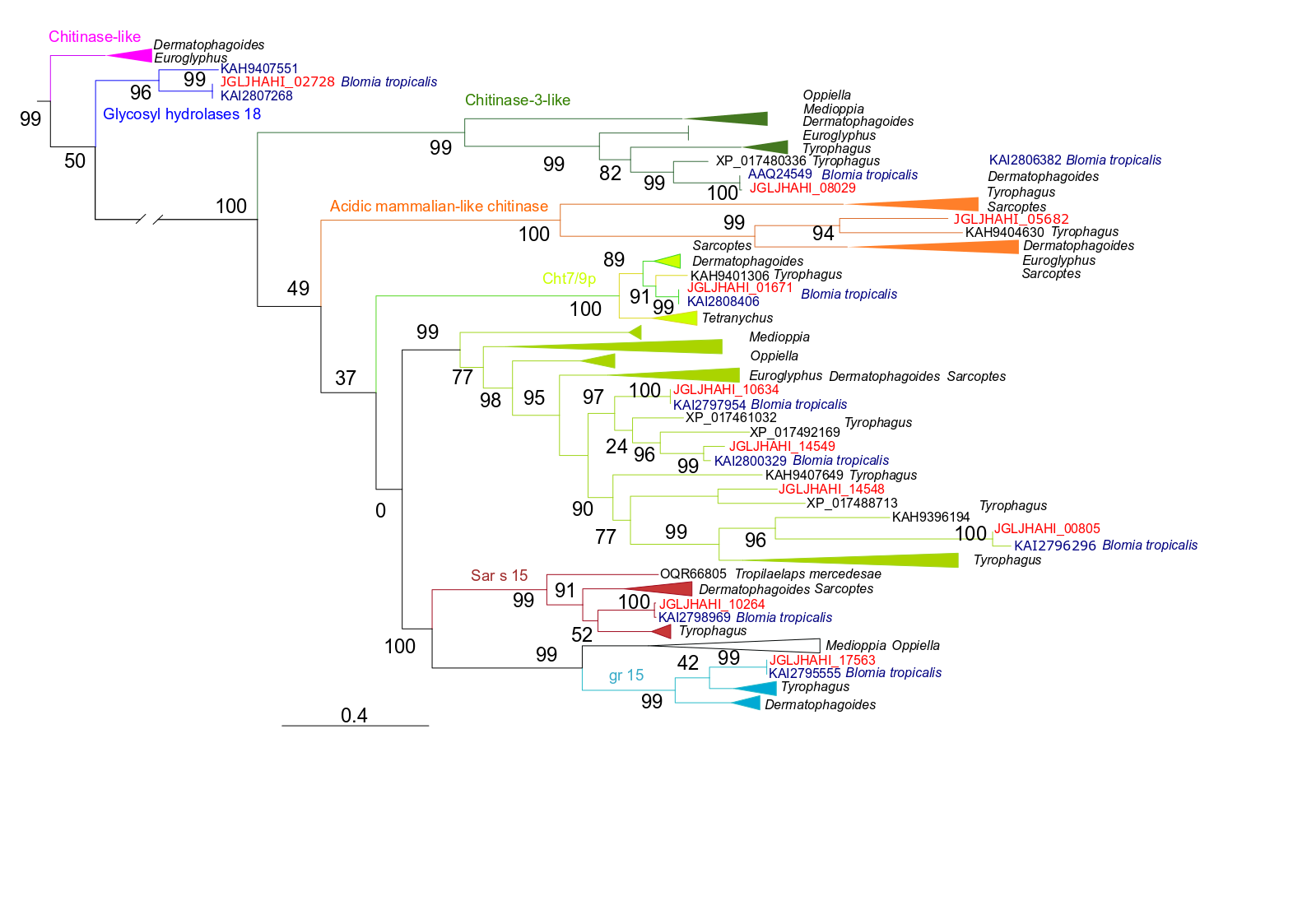


**Fig. S9A** Clustal alignment of a *Pseudomonas aeruginosa* (UniProt access A0A2R3IMN2) chitinase and the predicted *Blomia tropicalis* chitinase JGLJHAHI_02728 from the glycosyl hydrolase 18 cluster.

**Fig. S9B** Clustal alignment of a *Monascus purpureus* (UniProt acid A0A507QLV2) chitinase and the predicted *Blomia tropicalis* chitinase JGLJHAHI_02728 from the glycosyl hydrolase 18 cluster.

**Fig. S9C** Clustal alignment of an *Aspergillus terreus* (UniProt acid Q0CL102) chitinase and the predicted *Blomia tropicalis* chitinase JGLJHAHI_02728 from the glycosyl hydrolase 18 cluster.

**Fig. S10A** Clustal alignment of a *Pseudomonas aeruginosa* (UniProt accession A0A2R3IMN2) chitinase and the predicted *Blomia tropicalis* chitinase JGLJHAHI_05682 from acidic mammalian-like chitinases.

**Fig. S10B** Clustal alignment of a *Monascus purpureus* (UniProt acid A0A507QLV2) chitinase and the predicted *Blomia tropicalis* chitinase JGLJHAHI_05682 from acidic mammalian-like chitinases.

**Fig. S10C** Clustal alignment of an *Aspergillus terreus* (UniProt accession Q0CL102) chitinase and the predicted *Blomia tropicalis* chitinase JGLJHAHI_05682 from acidic mammalian-like chitinases.

**Fig. S11A** Clustal alignment of a *Pseudomonas aeruginosa* (UniProt access A0A2R3IMN2) chitinase and the predicted *Blomia tropicalis* chitinase JGLJHAHI_01671 from the Ch7/9p cluster.

**Fig. S11A** continued

**Fig. S12B** Clustal alignment of a *Monascus purpureus* (UniProt acid A0A507QLV2) chitinase and the predicted *Blomia tropicalis* chitinase JGLJHAHI_01671 from the Ch7/9p cluster.

**Fig. S12B** continued

**Fig. S12C** Clustal alignment of an *Aspergillus terreus* (UniProt acid Q0CL102) chitinase and the predicted *Blomia tropicalis* chitinase JGLJHAHI_01671 from the Ch7/9p cluster.

**Fig. S12C** continued

**Fig. S13A** Clustal alignment of a *Pseudomonas aeruginosa* (UniProt access A0A2R3IMN2) chitinase and the predicted *Blomia tropicalis* chitinase JGLJHAHI_08029 from the chitinase-3-like cluster.

**Fig. S13B** Clustal alignment of a *Monascus purpureus* (UniProt acid A0A507QLV2) chitinase and the predicted *Blomia tropicalis* chitinase JGLJHAHI_08029 from the chitinase-3-like cluster.

**Fig. S13C** Clustal alignment of an *Aspergillus terreus* (UniProt acid Q0CL102) chitinase and the predicted *Blomia tropicalis* chitinase JGLJHAHI_08029 from the chitinase-3-like cluster.

**Fig. S14A** Clustal alignment of a *Pseudomonas aeruginosa* (UniProt access A0A2R3IMN2) chitinase and the predicted *Blomia tropicalis* chitinase JGLJHAHI_10264 from Sar s15.

**Fig. S14B** Clustal alignment of a *Monascus purpureus* (UniProt accession A0A507QLV2) chitinase and the predicted *Blomia tropicalis* chitinase JGLJHAHI_10264 from Sar s15.

**Fig. S14C** Clustal alignment of an *Aspergillus terreus* (UniProt acid Q0CL102) chitinase and the predicted *Blomia tropicalis* chitinase JGLJHAHI_10264 from Sar s15.

**Fig. S15A** ClustalW alignment of a *Pseudomonas aeruginosa* (UniProt access A0A2R3IMN2) chitinase and the predicted *Blomia tropicalis* chitinase JGLJHAHI_17563 from allergen group 15.

**Fig. S15B** Clustal alignment of a *Monascus purpureus* (UniProt acid A0A507QLV2) chitinase and the predicted *Blomia tropicalis* lJGLJHAHI_17563 from allergen group 15.

**Fig. S15C** Clustal alignment of an *Aspergillus terreus* (UniProt acid Q0CL102) chitinase and the predicted *Blomia tropicalis* chitinase and JGLJHAHI_17563 from allergen group 15.

**Supplementary references**

Farris JS (1972) Estimating phylogenetic trees from distance matrices. Am Nat 106(951):645–668. <https://doi.org/10.1086/282802>

Hubert J, Nesvorna M, Klimov P, Dowd SE, Sopko B, Erban T (2019b) Differential allergen expression in three *Tyrophagus putrescentiae* strains inhabited by distinct microbiome. Allergy 74(12):2502–2507. <https://doi.org/10.1111/all.13921>

Lefort V, Desper R, Gascuel O (2015) FastME 2.0: a comprehensive, accurate, and fast distance-based phylogeny inference program. Mol Biol Evol 32(10):2798–2800. <https://doi.org/10.1093/molbev/msv150>

Meier-Kolthoff JP, Goker M (2019) TYGS is an automated high-throughput platform for state-of-the-art genome-based taxonomy. Nat Commun 10(1):2182. <https://doi.org/10.1038/s41467-019-10210-3>

Meier-Kolthoff JP, Sarda Carbasse J, Peinado-Olarte RL, Goker M (2022) TYGS and LPSN: a database tandem for fast and reliable genome-based classification and nomenclature of prokaryotes. Nucleic Acids Res 50(D1):D801–D807. <https://doi.org/10.1093/nar/gkab902>
